# Enhancing the utility of exploratory ‘factors associated with’ studies in global mental health: the importance of context in Sub-Saharan Africa

**DOI:** 10.1186/s13033-026-00741-w

**Published:** 2026-09-27

**Authors:** Joan Abaatyo, Wilber Ssembajjwe, Emmanuel Kiiza Mwesiga

**Affiliations:** 1 Department of Psychiatry, King Ceasor University, Kampala, Uganda; 2 Department of Psychiatry, Mulago Hospital, Kampala, Uganda; 3 MRC/UVRI/LSHTM, Kampala, Uganda; 4 Department of Psychiatry, College of Health Sciences, Makerere University, Kampala, Uganda

**Keywords:** Global mental health, Exploratory analysis, Multivariable regression, Causal inference, Epidemiology

## Abstract

“Factors associated with” studies, exploratory multivariable regression analyses that do not begin with a prespecified primary exposure, have recently come under increasing criticism, with some arguing that they are methodologically weak and contribute little to scientific knowledge. Much of this criticism is justified. Many published studies in global mental health (GMH) rely on small samples, inconsistent adjustment strategies, automated variable-selection methods, and interpretations that imply causality where none can be established. These limitations deserve attention and should not be overlooked. However, abandoning exploratory association studies altogether would ignore the realities of research in many low- and middle-income countries and could further marginalize evidence from the Global South. In global mental health, particularly in Sub-Saharan Africa, limited data systems, poorly understood causal pathways, and resource constraints often make longitudinal and experimental studies difficult to conduct. In these settings, exploratory analyses remain an important starting point for identifying patterns, generating hypotheses, and informing future research. Indeed, many important advances in epidemiology have emerged through an iterative process of observation, exploration, and subsequent causal investigation. We argue that the central issue is therefore not the use of exploratory association studies, but how they are designed, analysed, and reported. Rather than discarding them, researchers should clearly present their findings as hypothesis-generating, avoid automated stepwise variable selection, justify adjustment decisions using directed acyclic graphs (DAGs), adhere to established reporting guidelines, and interpret results within their local context. This is not a call to lower methodological standards in the Global South. Instead, it is a call to apply rigorous methods in ways that are appropriate to the realities of under-resourced settings while strengthening local capacity for causal inference. Silencing exploratory research would risk reducing the visibility of evidence from precisely those settings where stronger representation in global mental health research is most needed. Enhancing the quality of exploratory research can help advance global mental health research, particularly in settings where relevant evidence remains limited.

## Introduction

In their recent article, Lewer et al. argue that “factors associated with” studies, observational analyses built on exploratory multivariable regression without a prespecified primary exposure, are methodologically flawed and should largely be abandoned [1]. Their critique highlights valid concerns, including inappropriate adjustment, multiple testing, and post-hoc causal interpretation [1]. We agree with much of the diagnosis but believe that the proposed prescription warrants consideration from a broader range of perspectives. Recommending that the field abandon these studies altogether overlooks both the historical trajectory of epidemiology and the practical constraints of global mental-health research, particularly in Sub-Saharan Africa (SSA).

It is also worth being explicit about why this discussion belongs in this journal rather than only in correspondence to BMJ Medicine, where Lewer et al.’s piece appeared [1]. Decisions about which risk factors get screened for, which populations get prioritized, and where scarce services get directed in under-resourced mental health systems are frequently built on exactly this kind of exploratory evidence, because the cohort infrastructure the confirmatory-methods literature assumes simply is not there across much of SSA [2–4]. How this evidence gets produced and judged is therefore a mental-health-systems question, not only a statistical one. That is why our aim here is less to rebut Lewer et al. than to build on their argument: we want to show how the methodological and reporting quality of exploratory studies in Global Mental Health (GMH) can be improved, so that mental health systems in low-resource settings can keep drawing on this evidence responsibly.

We argue that exploratory association studies still serve a real scientific function when they are carried out thoughtfully, reported transparently, and read in context, but only if the weaknesses Lewer et al. and others have documented are actually addressed, not brushed aside. Rather than methodological relics, they remain essential tools in settings where foundational knowledge is limited, causal structures are not well understood, and data infrastructures are still emerging.

## Exploratory association studies in scientific discovery

Much of epidemiology, and indeed much of science, has advanced through iterative cycles of observation, association, hypothesis generation, and causal refinement [5–7]. Before the advent of formal causal-inference frameworks, many foundational discoveries emerged from exploratory analyses: the association between smoking and lung cancer, hypertension and stroke, air pollution and respiratory disease, or sexual behaviour and HIV risk [8–11]. These studies preceded the causal models we now take for granted.

Mental health research followed a similar trajectory. Influential observations connecting childhood adversity to adult psychopathology [12], and socioeconomic disadvantage to depression [13], initially emerged from associative work rather than predefined causal models. These early studies did not claim causality; instead, they generated hypotheses strong enough to justify qualitative inquiry, longitudinal cohort work, and experimental designs [12, 13].

The distinction between exploratory (hypothesis-generating) and confirmatory (hypothesis-testing) research gets blurred in practice more often than it should [14, 15]. Even within biostatistics, researchers have recently pointed out that a lot of published work is presented as confirmatory when it is, underneath, exploratory, which suggests the fix is better labelling, not getting rid of exploratory work altogether [14, 16].

To dismiss exploratory studies as inherently flawed is therefore to misinterpret the role they play in the scientific process. Exploratory multivariable regression does not compete with causal inference; rather, it typically precedes and informs it, and, when conducted and reported carefully, can provide preliminary insights that may help guide subsequent research examining causal relationships [17].

## Context matters: evidence production in Sub-Saharan Africa

Lewer et al. do not state this explicitly, but in our reading their critique implicitly presupposes research conditions more characteristic of high-income countries: robust electronic health records, large cohorts, stable funding, and well-established analytic capacity [1]. These assumptions do not reflect the global reality.

In SSA, mental-health research operates within structural constraints: fragmented health information systems, limited availability of specialised datasets, scarcity of longitudinal cohorts, and chronic underfunding [2–4]. We should be careful here, though: these constraints do not automatically justify exploratory “factors associated with” analyses on their own. Small exploratory studies are supposed to accumulate, through replication and synthesis, into something more definitive, and being under-resourced is not by itself a methodological excuse [18, 19]. What we are arguing is narrower: when the causal structure linking an exposure to a mental-health outcome has simply never been described empirically, exploratory analysis is often the step that has to come first, before a later, better-resourced study, quasi-experimental or cohort, can even specify a defensible causal model [20].

Under these conditions, exploratory multivariable regression is not a methodological shortcut, it is often a viable empirical approach. Such studies enable researchers to: identify patterns of depression, anxiety, suicidality, and self-harm in populations rarely studied [3, 21]; understand how sociocultural and economic determinants interact with mental-health outcomes in African contexts [4]; detect high-risk groups in settings with limited access to care [21]; and generate locally grounded hypotheses rather than importing causal assumptions developed elsewhere [22, 23].

This is not just a hypothetical benefit. Exploratory and qualitative work mapping the social and economic correlates of depression in SSA communities [21], fed directly into the design of task-sharing psychological interventions now built into several countries’ mental health systems [22], a fairly concrete example of exploratory findings turning into something testable and policy-relevant, rather than sitting on a shelf. A related example comes from research among refugee and displaced youth in Kampala, Uganda. Initial cross-sectional analyses identified food insecurity, violence exposure, social support and other contextual factors as important correlates of depression [24]. Subsequent work in the same population examined these factors longitudinally, using repeated observations to assess whether associations with depression persisted over time, including across the COVID-19 pandemic period. Food insecurity and violence remained associated with greater depressive symptoms, while social support was protective [25]. This progression illustrates how exploratory cross-sectional findings can identify locally relevant candidate factors that warrant testing in stronger longitudinal designs, rather than being treated as causal conclusions in themselves.

Far from being research “waste,” these studies provide some of the only empirical windows into emerging mental-health challenges in the region. Abandoning them outright, rather than reforming how they are conducted and reported, would disproportionately silence evidence from the Global South and widen global inequities in knowledge production.

## Acknowledging real weaknesses in the existing literature

We are not going to pretend that most published “factors associated with” studies in GMH, SSA included, are well done. Small-underpowered samples, adjustment sets chosen without much justification, automated stepwise selection, and causal language that outruns the study design, these show up constantly, and not just in this region [26–28]. Lewer et al. make a fair point when they note that inaccurate or overstated findings from such studies can end up shaping clinical guidelines or policy in ways that leave patients worse off [1]. We agree that this risk is real. Exploratory analysis cannot be treated as permission to fit models without constraint or to make causal claims the data cannot support.

A few of Lewer et al.’s specific criticisms are worth engaging directly rather than nodding along to in the abstract. Model instability from automated selection procedures is well documented [26, 27]. Collider bias, where adjusting for a variable that is itself a downstream effect of both exposure and outcome creates a spurious association, is a specific, avoidable failure when adjustment sets are chosen without any reference to an underlying causal structure, even a rough one [29–31]. And selective reporting, where the many small analytic choices available to a researcher get used, deliberately or not, to land on a significant result, is a documented threat to validity in its own right [28]. So our defense of exploratory “factors associated with” studies comes with a condition attached: it applies to studies that are framed honestly, run carefully, and interpreted modestly-not to the weaker examples that have, fairly, drawn criticism.

## Real problems, practical solutions

Addressing the concerns raised by Lewer et al. requires concrete changes in how these studies are designed, conducted, and reported, rather than simply calling for greater “transparency.” We therefore propose a set of practical standards for GMH and SSA researchers conducting exploratory “factors associated with” studies. Among these, four should be considered the non-negotiable minimum: explicitly framing findings as hypothesis-generating, developing a provisional DAG before selecting covariates, avoiding automated variable-selection procedures, and reporting all variables that were tested. The remaining practices build on this minimum and should be adopted where feasible to further strengthen methodological rigor and transparency:

### Say plainly that it is hypothesis-generating:

State in the abstract and conclusion that the associations reported are preliminary and hypothesis-generating, even when they are statistically significant. Do not leave readers to infer causal or clinical meaning that is not there [1].

### Sketch a DAG even before it is fully worked out:

Draw a provisional directed acyclic graph from whatever domain knowledge is available before choosing covariates. It is not perfect, but it disciplines adjustment decisions and helps avoid conditioning on a collider or mediator by accident, and it can be updated as understanding improves [30, 31].

### Skip automated variable selection:

Stepwise and similar procedures are known to produce unstable models that do not generalize. Choose covariates based on theory or prior evidence instead [26, 27].

### Report every variable that was tested, not only the ones that made the final model:

Include the full list of exposures and covariates considered, and explain the reasoning behind adjustment decisions. This cuts down on analytic flexibility and false positives [28].

### Register the analysis plan where feasible:

Even exploratory work benefits from registering the plan, dataset, and intended outputs in advance, the Open Science Framework or a local ethics-committee registry both work [32]. This does not require naming a single primary exposure, but it does limit how much can shift once the data are in hand.

### Follow the reporting guidelines that already exist:

STROBE for observational studies [33], and watch for the “Table 2 fallacy”, treating adjustment-variable coefficients as though they carry the same causal weight as the exposure of interest [34].

### Triangulate with qualitative and clinical evidence:

Numbers read differently next to qualitative and clinical context, and that combination strengthens interpretation and aligns with good practice in global mental-health research [21, 22].

### Root findings in the local health system:

Interpret results with the data infrastructure and health-system context they came from clearly in view, especially in low-resource settings [2–4]

None of this removes the limits that come with exploratory analysis. But it goes a long way toward reducing the overclaiming and instability Lewer et al. are right to flag, while still letting researchers in low-resource settings contribute evidence.

## Context-appropriate rigor, not lower standards

We want to be clear about one thing: this is not an argument for looser standards in SSA or anywhere else that is under-resourced. Data scarcity and tight budgets explain why exploratory designs are often the only option available, they do not excuse doing those designs badly. What we are calling for is applying rigorous standards in a way that fits the setting, using the practices above, alongside real investment in training and infrastructure that give researchers in the region better access to causal-inference methods, larger cohorts, and stronger data systems [35]. Rigorous exploratory work now, paired with building toward confirmatory and causal designs over time, is roughly the path other parts of epidemiology have already walked, from association to explanation. There is no reason SSA mental-health research cannot follow the same path, given the chance.

## Conclusion

Exploratory association studies are not a substitute for causal inference, and they should not be presented as one. We also accept that a good share of the existing “factors associated with” literature in GMH does not meet the bar we have laid out here. Still, in global mental health, and in Sub-Saharan Africa especially, exploratory multivariable regression deserves to remain in the methodological toolkit when it is framed honestly as hypothesis-generating. Variable selection and model specification should be guided by at least a basic theoretical or conceptual understanding of the hypothesized relationships among exposures, outcomes, confounders, mediators, and colliders. Findings should also be reported in accordance with established guidelines. Done that way, these studies raise the right questions, surface patterns that would otherwise go unnoticed, and build the groundwork that more rigorous causal research can stand on later. Rigorous, causally informed research is very often built on a foundation of exploratory work. Sub-Saharan African mental-health research may be following a trajectory similar to the one many now well-resourced settings once travelled, a trajectory along which contextually appropriate approaches to causal inference can be developed and refined to reflect local health systems, cultures, and research environments. Getting rid of exploratory multivariable regression altogether, rather than fixing how it is used, would foreclose that trajectory: it would mute research from low-resource settings, at a cost to global equity in mental-health evidence, before that research has had the chance to mature. What is needed is not fewer exploratory methods, but more disciplined, transparent, and context-aware use of the ones we already have.

## Data Availability

No datasets were generated or analysed during the current study.
